# Accuracy of heart girth tapes in the estimation of weights of pre‐weaned calves

**DOI:** 10.1002/vro2.16

**Published:** 2021-08-07

**Authors:** Virginia Sherwin, Robert Hyde, Martin Green, John Remnant, Emily Payne, Peter Down

**Affiliations:** ^1^ School of Veterinary Medicine University of Nottingham Leicestershire UK

**Keywords:** calves, dairy cattle, herd health, husbandry

## Abstract

**Background:**

Heart girth tapes (HGTs) are often used as an alternative to weight scales for calves. This study investigated the accuracy of HGT in estimating bodyweight and daily liveweight gain (DWLG) of pre‐weaned calves, and the impact of inter‐observer variation.

**Method:**

In Study 1, 119 calves were weighed using HGT and electronic scales on multiple occasions. Mixed‐effects models for both bodyweight and DLWG were used to determine the accuracy of HGT compared to the electronic scales. Simulation data were used to further analyse the accuracy of DLWG estimation including for factors such as the effect of group size on group DLWG estimates.

In Study 2, 10 observers weighed 20 pre‐weaned calves, using HGT and electronic scales. Mixed‐effect model was used to investigate the impact of different observers on the accuracy of HGT on measuring bodyweights.

**Results:**

Mixed‐effects model results suggest HGT provides a relatively accurate estimation of weight (MAE: 2.66 kg) and relatively inaccurate estimation of DLWG (MAE 0.10 kg/d). Simulated data identified associations between time between weight dates and error in DLWG estimation, with MAE of individual DLWG estimation decreasing from 0.43 kg/d when 14 days apart to 0.08 kg/d when 70 days apart. Increased calf numbers reduced error rates of group DLWG estimation, with <0.05 kg/d error achieved in >90% of simulations when 12 calves were weighed 70 days apart.

**Conclusions:**

HGTs are relatively accurate at estimating individual bodyweights but are unreliable methods for measuring DLWG in individual calves, particularly weighed within a short‐time period. Estimates at group level however are relatively accurate, providing there is a suitable period of time between weigh dates and an appropriate number of calves per group.

## INTRODUCTION

Heifer rearing represents a significant financial investment to dairy farmers, costing on average £1,391 on farms in the United Kingdom.^1^ Feed costs represent a significant financial input, which may be affected by the desired target daily liveweight gain (DLWG) for the heifer. Factors affecting the DLWG of heifers and the use of different management decisions to maintain target DLWG while reducing the cost per kg of gain have been the subject of a growing amount of research, especially the pre‐weaning period where the cost is on average the highest at £3.14 per day on UK farms.^2^ Studies have also highlighted there are additional health and production benefits associated with growing at the target DLWG of 0.8 kg/day in the pre‐weaning period, for example, the average DLWG in the first 2 months of life has been linked to survival to the end of the second lactation and increased milk production.^3^, ^4^, ^5^

The accurate weighing of pre‐weaned calves is important for measuring the growth rate of calves. Estimates are commonly used on farms for management purposes, as well as for determining the dosage of medications, including antibiotics and anthelmintics. Multiple studies have reported variation in the abilities of farmers and veterinary surgeons to accurately estimate the bodyweight of cattle visually, with the majority of people underestimating the bodyweight of the animals,^6^, ^7^ which could lead to an increased risk of antibiotic and anthelmintic resistance developing.^8^, ^9^ Other studies have reported that there is a tendency to overestimate the bodyweight of animals which are <150 kg when visual estimates were compared to heart girth tape (HGT) estimates,^10^ which could result in inappropriate management, in terms of groupings and nutrition.

The gold standard method of weighing calves involves the use of a calibrated weight scale or weighbridge, both of which involve considerable financial investment from the farmer and will often result in a non‐portable system. This has led to the development of other more practical methods being used on farms, including HGT. A recent study highlighted that many pre‐weaned dairy calves had very low growth rates, despite having a high feed conversion efficiency at this age and that monitoring heifer growth during the rearing period would help improve the efficiency of heifer rearing.^11^ Therefore, it is important to have a reliable and cheap method of estimating the bodyweight of pre‐weaned calves on‐farm.

The relationship between bodyweights obtained via electronic scales and HGT has been investigated previously with some studies reporting a poor correlation between the HGT and weigh scales,^12^, ^13^ and other studies reporting a good correlation.^14^ One of the limitations of these studies is that they have included heavier heifers which may have skewed the data. None of these studies have investigated the reliability of estimating DLWG using HGT, which most likely reflects the large sample size required for ensuring the reliability of the results. The use of simulation models allows research questions to be answered without requiring large numbers of animals^15^ and has been used in this study.

The aim of this study is to investigate the accuracy of DLWGs calculated from bodyweights estimated using HGT and weigh scales in pre‐weaned calves, as well as the level of inter‐observer agreement.

## MATERIALS AND METHODS

Two farms based in Leicestershire were selected for sampling calves. Farm A was a 350 cow spring block calving herd, with the heifers selected for the study being mostly Jersey crossbred calves. Farm B was a 300 cow all year‐round calving herd, with the predominant breed of youngstock being Holstein.

Data were collated in Microsoft Excel 2016, and statistical analysis was performed in R statistical software^16^ using the tidyverse package.^17^

### Study 1

All calves present on‐farm were weighed on multiple occasions using a HGT (Weight Measuring Tape for Cattle and Pigs, Rondo) by Observer 1 between October 2018 and February 2019 at 2‐ to 3‐week intervals. The batch of calves was then weighed immediately using calibrated electronic weigh scales (Tru‐test Eziweigh 5i indicator, Border Software, Welshpool). The weigh scales were calibrated at each recording using a known weight. The breed and sex of each calf was recorded, as well as their date of birth. Calf breeds were categorised as Holstein or Holstein Friesian (HF), Jersey or Jersey cross (J) and Norwegian reds (NR).

Mixed effects models for both bodyweight and DLWG were created using the lme4 package.^18^ As individual calves were weighed multiple times, calf ID was included as a random effect, with both HGT measurement and breed as fixed effects as follows:

$$Y_{ij}=\mu +\beta_{1}X_{1ij}+\beta_{2}X_{2ij}+U_{j}+\in$$

Where $Y_{ij}$ is the weigh scale‐estimated DLWG of the *i*th measurement from the *j*th calf. $X_{1ij}\;$represents HGT‐estimated DLWG for the *i*th measurement of the *j*th calf, with breed represented by $X_{2ij}$. μ represents the intercept, $U_{j}$ as the calf‐specific random effect for the *j*th calf and ∈ as the random error. The assumed distributions of U and ∈ are normal, with mean zero. Calf age and the time between weaning (d) were also added to the model and were retained if model performance (assessed by mean absolute error) was improved.

To further investigate the accuracy of DLWG estimation at a range of weights and calf numbers, a simulated dataset (SIM) was created, where data were simulated as follows. Ten thousand calves were simulated, with two bodyweights for each calf. Ages between the two bodyweights were randomly sampled from a uniform distribution between 10 and 70 days. Breed of each calf was simulated by randomly sampling HF, J and NR in equal proportion to the original dataset (0.53, 0.29 and 0.18)

True birthweight was simulated from a normal distribution, with mean 40 kg for HF calves and SD of 4.8 kg.^11^ In the absence of published data for GB calves, birthweights for J and NR calves were estimated as 30 kg, with SD 4.8 kg. True DLWG were simulated for each calf by randomly sampling from a normal distribution with mean and SD from weigh scale‐estimated DLWG from study 1 (0.76 and 0.37). True second bodyweight was calculated as birthweight plus the age of the calf multiplied by the true DLWG.

The error in HGT bodyweight estimation was simulated by randomly sampling from a normal distribution, with mean (using the mean absolute error) and SD (standard deviation) calculated from residuals after removing random effects (only including fixed effects) from the mixed effects model for bodyweight. The HGT estimate of birthweight was calculated from the true birthweight randomly plus or minus the error generated for birthweight. The HGT estimate of the second bodyweight was calculated from the true second bodyweight randomly plus or minus the error generated for the second bodyweight.

HGT estimated DLWG was calculated by dividing the difference in bodyweights by the difference in age for HGT bodyweights, and the error in HGT‐estimated DLWG was calculated as the HGT estimated minus the true DLWG. The effect of age between weights on the accuracy of individual HGT‐estimated DLWG was compared with true DLWG.

To analyse the effect of group size on group DLWG estimate, the sampling procedure was repeated, but only including five categories of ages between weights (14 days, 28 days, 42 days, 56 days and 70 days sampled with uniform distribution). This procedure was repeated 1000 times, resulting in 1000 datasets of 10,000 calves. The mean number of calves required to achieve <0.05 kg/d error between HGT and true group DLWG was calculated for each age category across all 1000 datasets. The number of calves required to achieve <0.05 kg/d error between HGT and true group DLWG in 90%, 95% and 99% of the 1000 datasets was also calculated. The effect of both age between weights and group size for HGT‐estimated group DLWG was compared with true group DLWG.

### Study 2

To investigate the inter‐user variability when using HGT, 20 pre‐weaned Holstein calves from Farm B, of varying ages, were weighed using a Rondo weight tape by 10 different observers. The observers consisted of six farm animal veterinarians based at the University of Nottingham and four final year undergraduate veterinary students from the University of Nottingham, who were undertaking their farm animal rotation on the day of sampling. The calves were weighed using a calibrated electronic weigh scales at the end of the weighing session.

Light's and Fleiss’ kappa were used to investigate correlations between observers using the irr package.^19^ A mixed effects model was created using the lme4 package^18^ with observer as a random effect and HGT as a fixed effect. Variance was explored to determine the variation explained at observer level, and residuals were examined to ensure model fit.

## RESULTS

### Study 1

A total of 354 bodyweights were obtained from 146 calves, with calves being weighed between 1 and 4 times per calf. These animals varied in age from 1 to 90 days old, with a range of bodyweights from 30 to 151 kg. In terms of numbers of calves weighed at different ages, 13% and 19% of the weights occurred at 15–21 days and 22–28 days old, representing that midway part of the pre‐weaning period. Eighteen per cent of the weights were taken around the time of weaning (50–77 days). Calves on Farm A were predominantly Jersey x Friesian crossbreds (J, *n* = 67) and Norwegian Red (NR, *n* = 21) with calves on Farm B being predominantly Holstein or Holstein‐Friesian calves (HF, *n* = 70). A small number of dairy x beef calves (*n* = 22 calves) were present. There were 135 female calves and nine male calves. Male dairy and beef cross calves (*n* = 27) were removed from the dataset resulting in 298 bodyweights from 119 calves in the final dataset, with 188, 68 and 42 bodyweights from 64, 34 and 21 calves for HF, J and NR calves, respectively.

**FIGURE 1:**
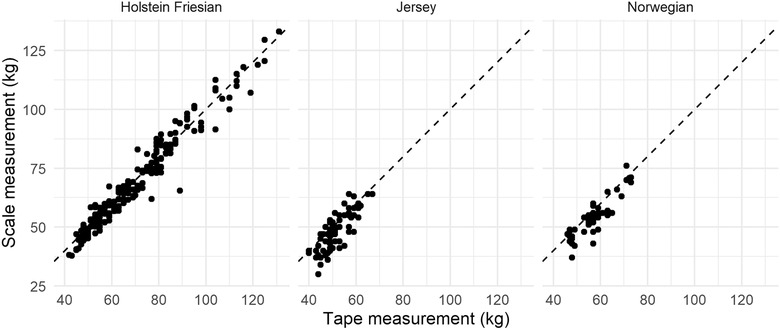
Relationship between heart girth tape and electronic scale measurement of bodyweight (kg) for Holstein Friesian, Jersey and Norwegian calves

There appeared to a good correlation between HGT and weigh scale measurements of bodyweight (Figure 1). A significant association of both breed and HGT bodyweight with weigh scale bodyweight was found using the mixed effects model (Table 1) with a coefficient of 1.00 kg (95% CI 0.97–1.04) for HGT measurement and a coefficient of −2.46 kg (95% CI −4.03–0.90) and −2.42 (95% CI −4.19–0.66) for J and NR calves, respectively. Age of calf was also added as a fixed effect during model building, but as it did not improve model performance, this variable was not included in the final model. The R^2^ for this model was 0.97 with an MAE of 2.66 kg. When only using fixed effects (and excluding the random effect of calf), the R^2^ was 0.95, with an MAE of 3.41 kg.

**TABLE 1:** Results from mixed effects model predicting individual bodyweight measured by electronic scales

Term	Coefficient (95% CI)	*p*‐value
(Intercept)	−1.04 (−3.52–1.44)	
Tape	1.00 (0.97–1.04)	<0.001
Breed Jersey (Ref: Holstein Friesian, HF)	−2.46 (−4.03–0.90)	0.002
Breed Norwegian Red (Ref: HF)	−2.42 (−4.19–0.66)	0.008

**FIGURE 2:**
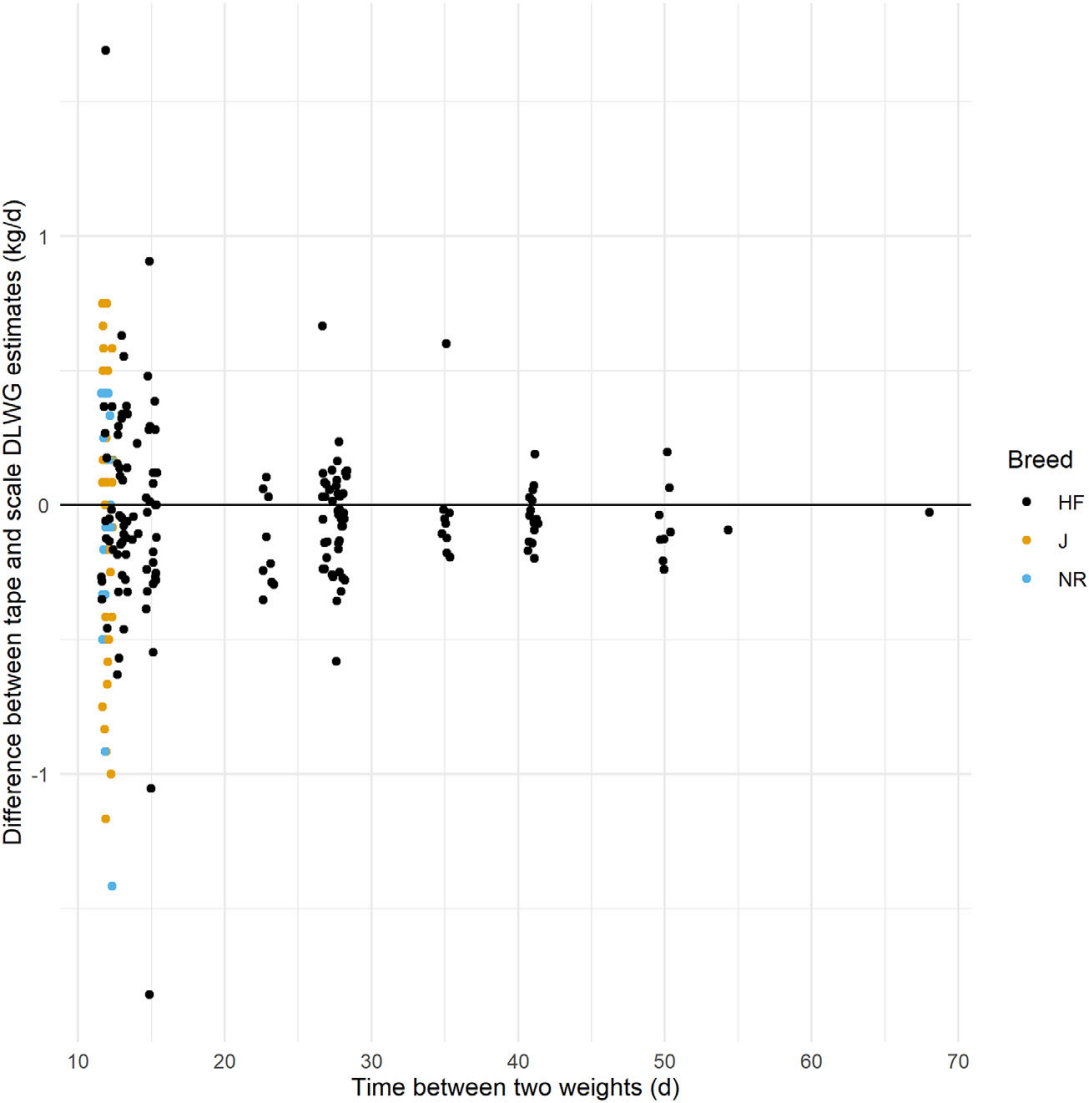
Errors in estimation of the daily live weight gain (DLWG) using a heart girth tape weight for different breeds and for varying time points (in days) between two weight estimates. HF: Holstein Friesian; J: Jersey; NR: Norwegian Red

**FIGURE 3:**
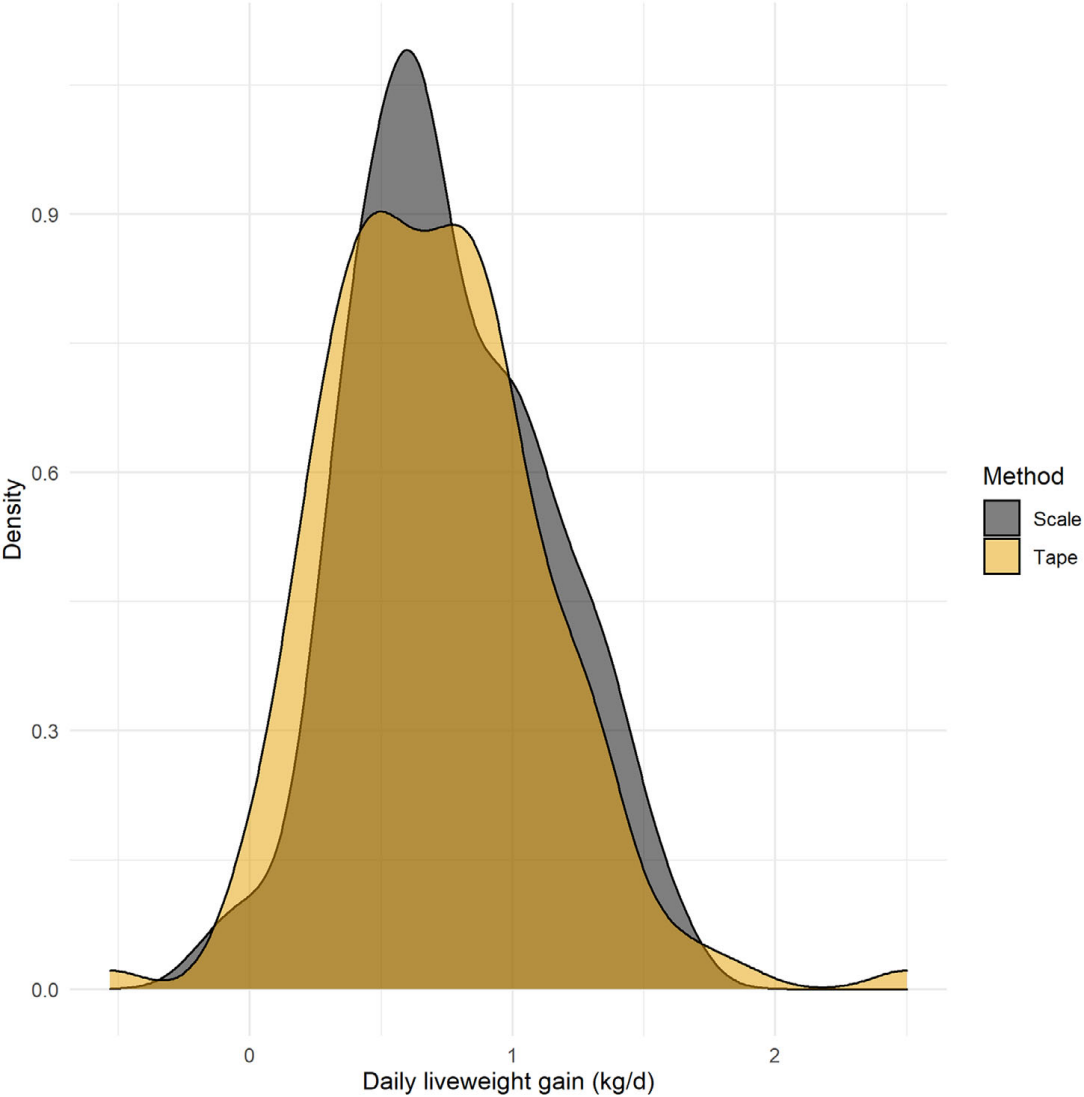
A density plot of the daily liveweight gains (DLWG) of the calves in the study using an electronic scale measurement and a heart girth tape method

The mean weigh scale‐measured DLWG for all calves was 0.76 kg/d (0.75 kg/d, 0.74 kg/d and 0.86 kg/d for HF, J and NR calves respectively), as shown in Figure 2. Mixed effects model performance using breed, tape‐estimated DLWG, calf age at weighing and age difference between weights resulted in an MAE of 0.10 kg/d and R^2^ of 0.82, although model performance decreased to an MAE of 0.20 kg/d and R^2^ of 0.34 when only fixed effects were included, and analysis of residuals suggested relatively poor model fit. The accuracy of DLWG estimation was reduced at younger ages (Figure 3), and model performance was decreased when filtering to only include DLWG estimates with <21 days between bodyweights (MAE of 0.25 kg/d and R^2^ of 0.28).

**FIGURE 4:**
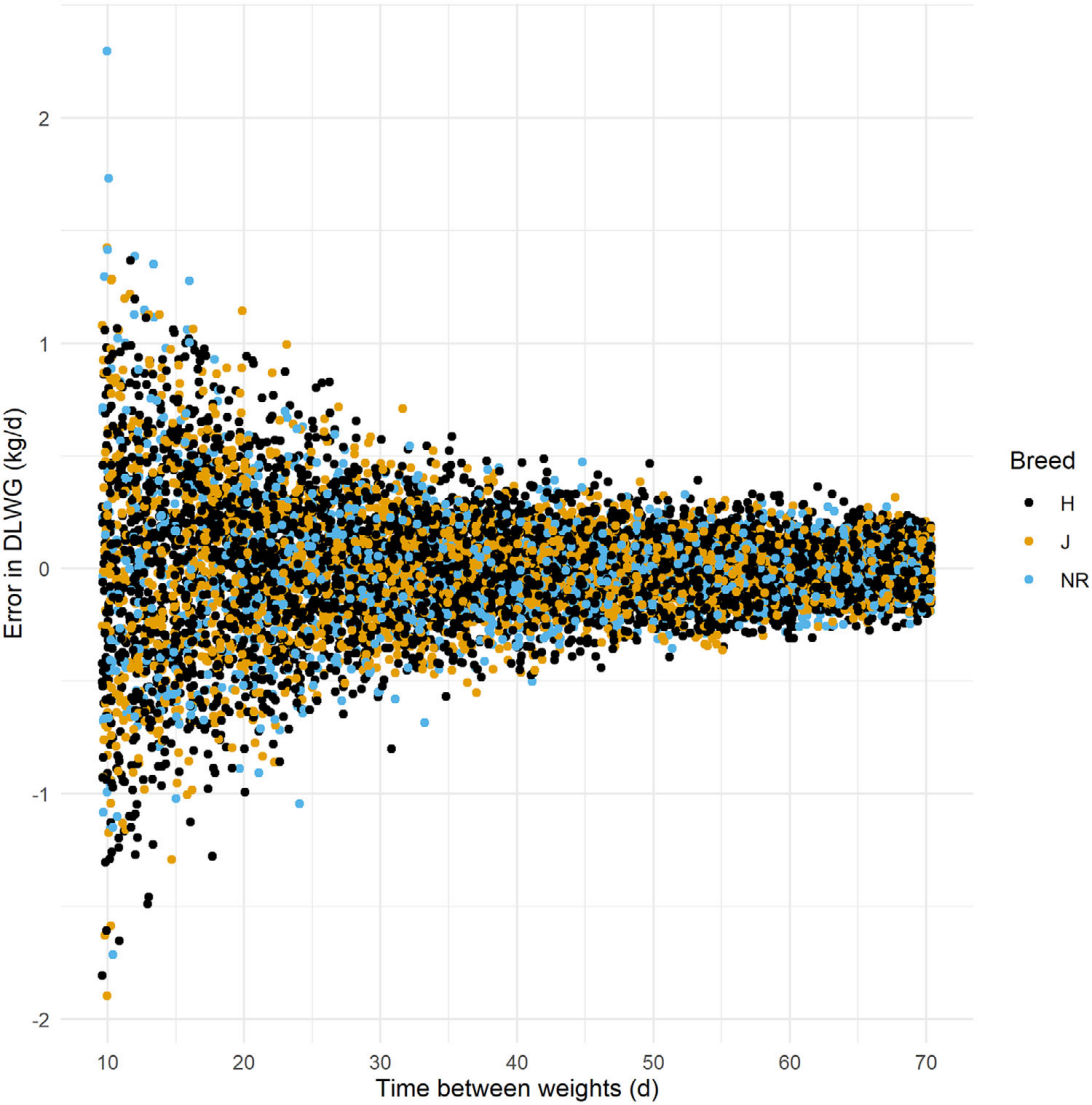
Mean absolute error in daily liveweight gain (DLWG) using a heart girth tape method, estimate by time between weighings (in days) and by breed, using 10,000 simulated calves. HF: Holstein Friesian; J: Jersey; NR: Norwegian Red

Of the 10,000 calves simulated in the SIM dataset, error rates between HGT estimations and true DLWG were similar to those in the 'real' dataset (Figure 3), and the absolute error in DLWG estimation had an asymptotic association with time between weight dates (Figure 4), with MAE in individual DLWG ranging from 0.43 kg/d when measured 14 days apart to 0.08 kg/d when 70 days apart.

**FIGURE 5:**
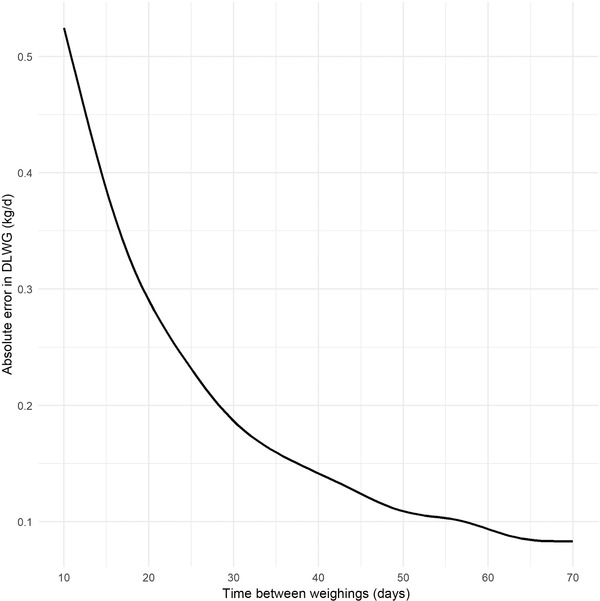
Mean absolute error in group daily liveweight gain (DLWG) estimation by time between weighings and number of calves in group

Of the 1000 repeated simulations of 10,000 calves, error rates between HGT estimation and true DLWG at group level were dependent on both group size, and the interval between weigh dates (Figure 5). The mean number of calves required to achieve <0.05 kg/d absolute error rate for estimation of DLWG at group level ranged from 67 calves at 14 days between weigh dates, to three calves at 70 days between weigh dates. To achieve <0.05 kg/d absolute error between HGT estimated and true DLWG at group level in >90% of simulations, 264 calves would be required if weighed with a 14 days interval between weights, with 12 calves required if weighed with a 70 days interval between weights (Table 2).

**TABLE 2:** Number (*N*) of calves required to achieve a 0.05 kg/d absolute error rate in estimating group daily liveweight gain by heart girth tape over 1000 repeats of 10,000 simulated calves on average (mean) and in 90%, 95% and 99% repeats

		Number (*N*) of calves required to achieve <0.05 kg/d absolute error between heart girth tape estimation and true daily liveweight gain in a given proportion (%) of simulation repeats		
Age between two weighings	Mean number of calves required	90%	95%	99%
14	67	264	378	604
28	18	66	88	167
42	7	31	42	74
56	4	18	26	40
70	3	12	16	27

### Study 2: Inter‐user variability

**FIGURE 6:**
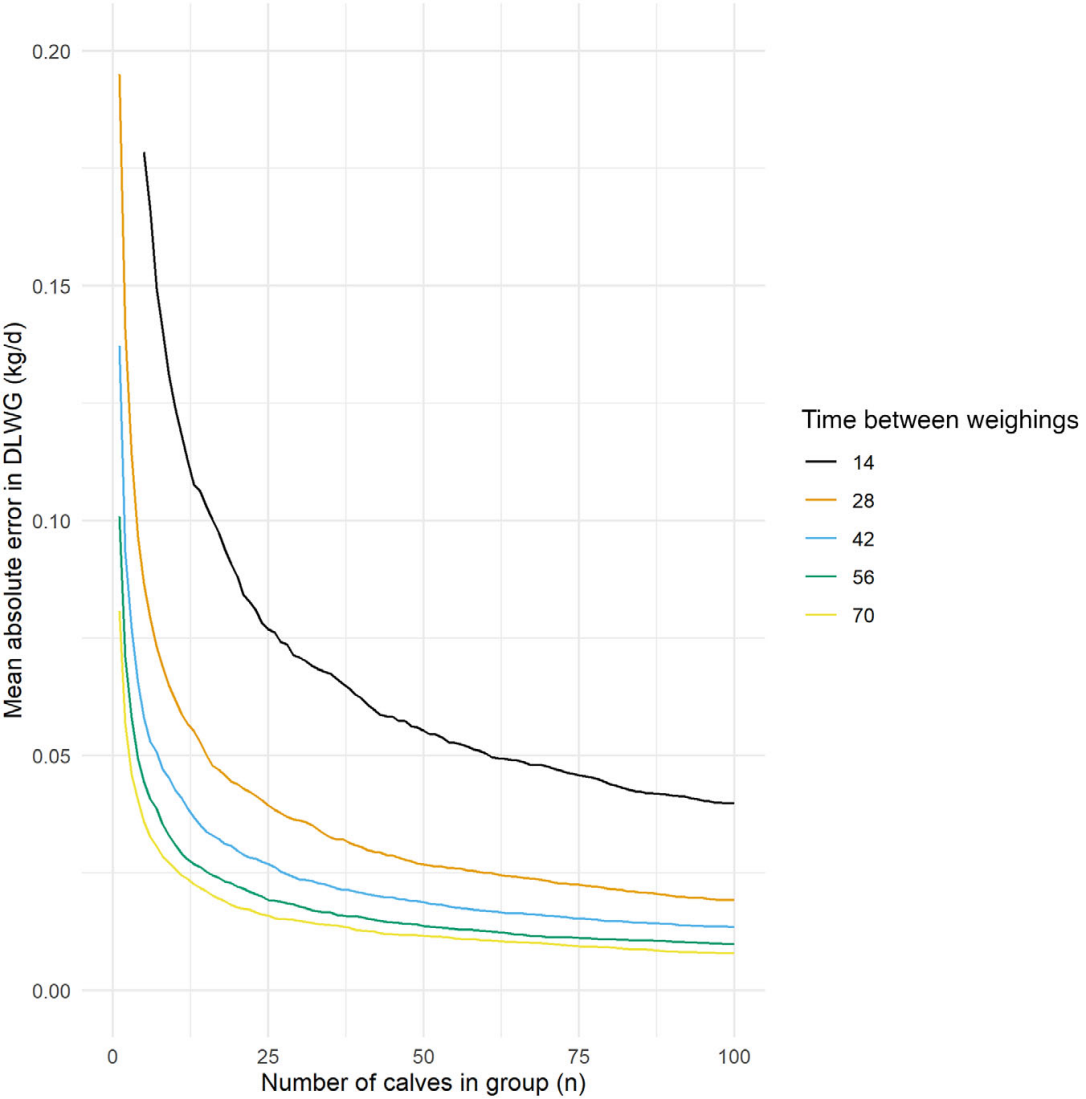
Mean error in heart girth tape daily liveweight gain (DLWG) by time between weighings in days using 10,000 simulated calves

Correlation between users for individual bodyweights were 0.102 and 0.0971 for Light's and Fleiss’ Kappa, respectively. Mixed effects model suggested HGT bodyweight was associated with weigh scale bodyweight (0.94 kg, 95% CI 0.91–0.97) with an R^2^ of 0.955, with a MAE of 3.90 kg. The analysis of variance from this model suggested that 32.42% of variation was at the level of observer, after accounting for the fixed effect of the HGT. Visual analysis suggests that error for individual operators was relatively consistent (Figure 6).

## DISCUSSION

**FIGURE 7:**
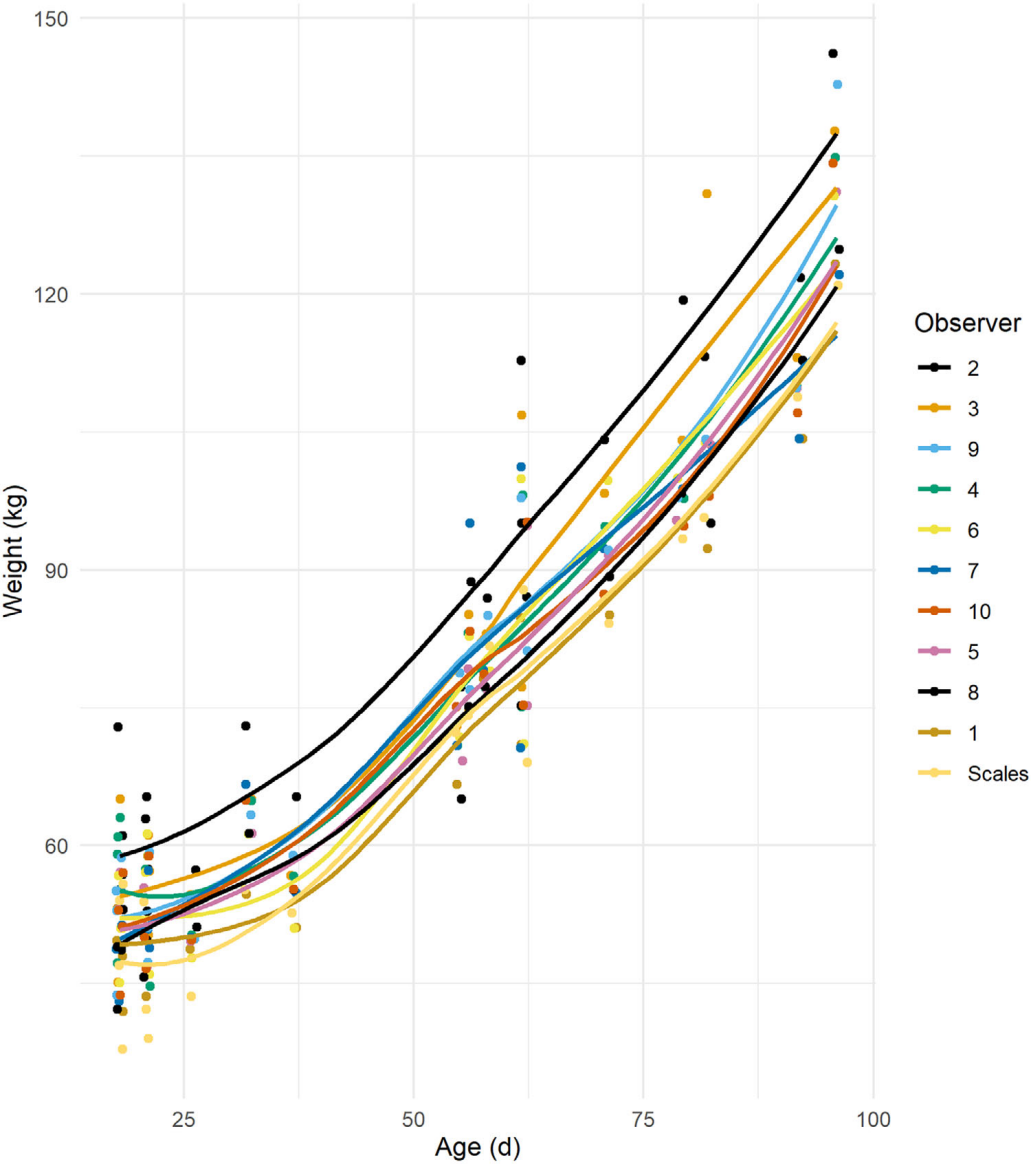
Bodyweight by age (in days) using a heart girth tape method for 10 observers compared with electronic scale weight for 20 pre‐weaned calves

This study shows that the estimation of DLWG for individual animals using HGT is likely to be relatively inaccurate, particularly when time between weights is relatively short (Figures 2 and 7). Analysis of the simulated data suggests that the absolute error in DLWG estimation by HGT is dependent on the interval between weigh dates, with the absolute error ranging from 0.43 kg/d when measured 14 dyas apart, decreasing to 0.08 kg/d when 70 days apart. While the error estimation of individual weights by HGT is relatively low, this can dramatically affect the estimation of DLWG, particularly when calves are weighed frequently. This study suggests that HGTs are a relatively unreliable method for measuring DLWG in individual calves, especially over a short‐time period.

The estimation of DLWG at group level is dependent on both the interval between weights and the number of calves measured. For example, the number of calves required to achieve a <0.05 kg/d error rate with HGT, with 90% confidence is 264 calves if calves are weighed with a 14 days interval between weights; however only 12 calves if weighed with a 70 days interval between weights (Table 2). While the estimation of individual DLWG from HGT is likely to be of limited use, the estimation of group DLWG by HGT can be relatively accurate, depending on the number of calves weighed and the length of time between weights.

Estimations of bodyweight by HGT are likely to be relatively accurate in the estimation of weights of pre‐weaned calves, with model results suggesting a mean absolute error of 2.66 kg, regardless of age. Age of calf was introduced to models to predict both individual weight and DLWG but did not result in any improvements in model performance so were excluded from the model. This suggests that the reliability of HGT at an individual calf level for body weight is relatively accurate. These findings differ from other studies, which indicated a poor correlation between HGT and weigh scale measurements for bodyweight in calves < 3 months old (*n* = 32 calves).^12^ Previous studies report less than 8% difference between real and predicted weights for 26 observers weighing 26 heifers between 50–550 kg, which was the equivalent of 4–8 kg difference for heifers < 150 kg (*n* = 3 heifers).^14^ Of the variation in bodyweights measured using HGT in study 2, 32.42% was at the level of the observer (MAE 3.9 kg). The number of calves available was limited at the time of measurement, and although it is possible that repeating the trial with increased numbers might provide a higher degree of accuracy in the calculation of agreement between observers, a relatively large proportion of the variation in weight was explained by the observer. It is recommended that the operator should remain consistent when weighing calves for DLWG, especially as it visually appears that the error between observers remains relatively consistent (Figure 5).

It is not clear how generalisable this research is to the rest of the UK as only two farms were included in this study. The calves included in this study were limited in terms of age, breed and sex, and caution should be exercised in extrapolating these results beyond these. Only one type of HGT was used during both studies and the results may not, therefore, be as applicable to other HGTs. However, it is likely that the findings regarding DLWG would be applicable to other HGTs, providing the same HGT is used each time bodyweights are taken. The accuracy of DLWG estimation by age and by group size is largely a mathematical question and the number of calves available through manual weighing of calves would be a significantly limiting factor in data exploration. Simulation modelling was therefore used in this study to provide a larger representative sample size based on our initial dataset to analyse HGT‐estimated DLWG. A potential limitation of the simulation methodology was the assumption of a linear growth curve between first and second weights. While a non‐linear growth curve might be expected in the pre‐weaning period,^11^ the error rates between tape and scale estimations of either weight or DLWG will be unaffected in the current simulation as age was not found to significantly affect weight estimates of tape (Table 1). The standard deviation of birthweights for J and NR calves was assumed to be the same as HF calves, although it is possible that variations in birthweight SD may exist that could not be fully analysed in the current study due to limited numbers of J and NR calves. While a potential limitation of this approach was the assumption of a normal distributed error, visual analysis of the data in Figure 3 would suggest this is not an inaccurate assumption and the use of simulated data in this study allows for a robust estimate of error for both individual or group estimation of DLWG at a variety of time points that would otherwise be both expensive and time consuming to achieve.

## CONCLUSIONS

The results from this study suggest that while HGTs are relatively accurate at estimating individual bodyweights, they are relatively inaccurate at estimating DLWG for individual animals, particularly when weighed within a short‐time period. However, DLWG estimates at group level are likely to be relatively accurate, providing there is a suitable period of time between weigh dates and an appropriate number of calves per group, for example weighing a cohort of 12 calves at 70‐day intervals. Practitioners and farm advisors should ensure an adequate number of calves are weighed depending on the time between weighing to provide a suitably accurate estimation of DLWG at group or farm level.

## CONFLICTS OF INTEREST

The authors declare that there are no conflicts of interest that could be perceived as prejudicing the impartiality of the research reported.

## ETHICS STATEMENT

Ethical approval for this work was granted by the Research Ethics Committee at the School of Veterinary Medicine and Science, University Of Nottingham.

## AUTHOR CONTRIBUTIONS

Virginia Sherwin conceived and planned the studies. Virginia Sherwin, Emily Payne and John Remnant carried out Study 1 and Study 2, with Peter Down being involved with Study 2. Robert Hyde, Peter Down and Martin Green planned and carried out the statistical analysis, including the simulation modelling. All authors contributed to the interpretation of the results. Virginia Sherwin took the lead in writing the manuscript, with a large input from Robert Hyde. All authors provided critical feedback and helped shape the research, analysis and manuscript.
